# Internalized Tau sensitizes cells to stress by promoting formation and stability of stress granules

**DOI:** 10.1038/srep30498

**Published:** 2016-07-27

**Authors:** Cecilia A. Brunello, Xu Yan, Henri J. Huttunen

**Affiliations:** 1 Neuroscience Center, University of Helsinki, Finland

## Abstract

Stress granules are membrane-less RNA- and RNA-binding protein-containing complexes that are transiently assembled in stressful conditions to promote cell survival. Several stress granule-associated RNA-binding proteins have been associated with neurodegenerative diseases. In addition, a close link was recently identified between the stress granule core-nucleating protein TIA-1 and Tau. Tau is a central pathological protein in Alzheimer’s disease and other tauopathies, and misfolded, aggregated Tau is capable of propagating pathology via cell-to-cell transmission. Here we show that following internalization hyperphosphorylated extracellular Tau associates with stress granules in a TIA-1 dependent manner. Cytosolic Tau normally only weakly interacts with TIA-1 but mutations mimicking abnormal phosphorylation promote this interaction. We show that internalized Tau significantly delays normal clearance of stress granules in the recipient cells sensitizing them to secondary stress. These results suggest that secreted Tau species may have properties, likely related to its hyperphosphorylation and oligomerization, which promote pathological association of internalized Tau with stress granules altering their dynamics and reducing cell viability. We suggest that stress granules and TIA-1 play a central role in the cell-to-cell transmission of Tau pathology.

The human genome encodes at least 1500 RNA binding proteins (RBPs) that regulate RNA metabolism from biogenesis to transport, localization and degradation, therefore playing a crucial role in cellular homeostasis^1^,^2^. Remarkably, many genetic alterations in RBP-coding genes have been associated with neurodegeneration. For example, mutations in fused in sarcoma protein (FUS), Tar DNA-binding protein 43 (TDP-43) and heterogeneous nuclear ribonucleoproteins (hnRNPA1/hnRNPA2B1) alter their localization or promote aggregation, and have been linked to amyotrophic lateral sclerosis (ALS) and frontotemporal dementia (FTD)^3^,^4^,^5^,^6^,^7^. Other motor disorders caused by mutations in RBPs include spinocerebellar ataxia-2, caused by expanded glutamine repeats in Ataxin-2 gene^8^, mutations in survival motor neuron protein (SMN) linked to spinal muscular atrophy^9^, and a mutation in TIA-1 linked to Welander distal myopathy^10^. In addition, cognitive impairment can be caused by mutations in RBPs, as is the case with mutations in the gene coding the Fragile X mental retardation protein (FMRP), which can cause a variety of cognitive deficits ranging from congenital mental retardation to inherited autism^11^.

A common characteristic for many RBPs is their involvement in stress granule (SG) formation or function. SGs are RNA granules that transiently assemble in stressful conditions to promote cell survival by blocking translation of non-essential mRNA’s and by sequestering pro-apoptotic proteins^12^,^13^. Interestingly, several studies have reported the presence of SG markers in pathological inclusions of several neurodegenerative disorders^14^. Also, mutations in the valosin-containing protein (VCP) gene, associated with clearance of stress granules, cause autosomal dominantly inherited ALS^15^,^16^ suggesting that disturbances in RNA metabolism and SG dynamics are involved in the pathogenesis of neurodegenerative diseases.

A SG marker and nucleating protein TIA-1 has also been found in Alzheimer’s disease neurofibrillary tangles, composed of hyperphosphorylated and aggregated Tau, in increasing amounts with increasing disease severity^17^. Currently, little is known about the relationship between Tau and stress granules. Moreover, despite many reports that indicate cell-to-cell transmission of pathological Tau species and seeding to promote degeneration (recently reviewed in^18^), the cellular mechanisms of this phenomenon remain poorly understood. In particular, how exogenous Tau accesses cells is still controversial; bulk endocytosis^19^, macropinocytosis^20^ and permeabilization of the membrane following Tau interaction with lipid rafts^21^ have been proposed. In this study, we show that secreted Tau is localized to cytosolic stress granules after internalization. Our current results suggest that, differently from normal cytosolic Tau, internalized extracellular Tau associates with SGs, interferes with their normal function and turnover, and reduces viability of the recipient cells. TIA-1 appears to play a central role in the recruitment of Tau to SGs.

## Results

### Internalized Tau is recruited to stress granules

**Figure 1:**
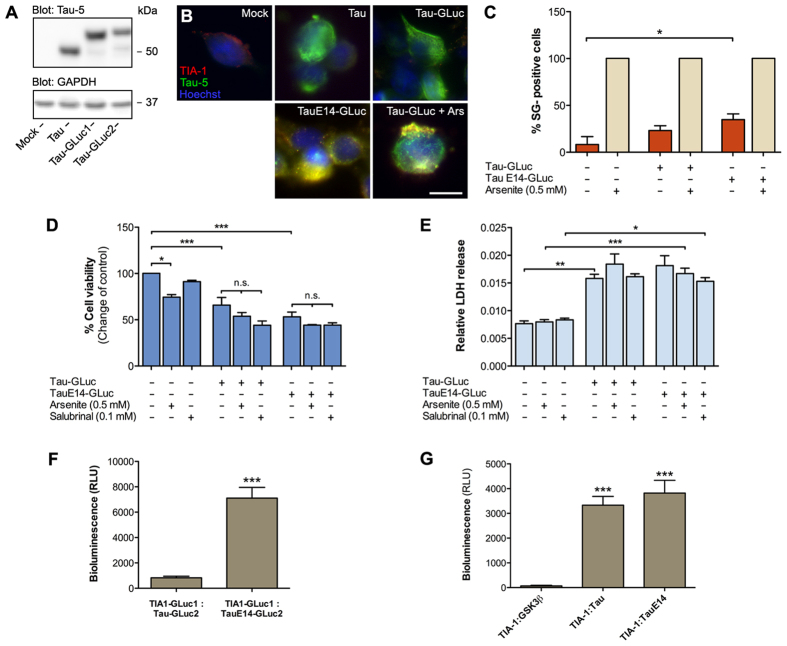
Transfected and internalized extracellular Tau differ in their ability to associate with stress granules. (**A**) Expression of non-tagged Tau, Tau-GLuc1/2 and TauE14-GLuc1/2 in HEK293T cells as detected by Western blot. The blot picture was cropped from a larger original image, maintaining all the stained bands. (**B**) HEK293T cells transiently transfected with the above-mentioned constructs and stained with Tau-5 (green) and TIA-1 (red) antibodies. Arsenite (0.5 mM for 30 min) was used as a positive control for induction of stress granules. (**C**) Quantitative analysis of stress granule formation. Stress granule-positive cells were counted among the Tau-transfected cells. Arsenite treatment promoted stress granule-formation in all cells while only some Tau-transfected, and more efficiently TauE14-transfected, cells contained stress-granules (n = 3). (**D**) Resazurin-based cell viability assay with HEK293T cells transiently transfected with the Tau constructs. Salubrinal and arsenite were used as positive controls for stress granule induction (n = 4). (**E**) LDH release assay in HEK293T cells transfected with the Tau constructs and treated with salubrinal and arsenite as positive controls (n = 4). Relative LDH release was calculated from the ratio of LDH in the media and total LDH (from media and the cells). (**F**) Protein-fragment complementation assay (PCA) in HEK293T cells transiently transfected with TIA-1-GLuc1, Tau-GLuc2 and TauE14-GLuc2 (n = 3). (**G**) PCA-based coplating experiment to study cell-to-cell transfer of Tau. Batches of HEK293T cells were separately transfected with a single PCA construct (TIA-1-GLuc1, Tau-GLuc2, TauE14-GLuc2 or GSK3β-GLuc2 as a control), followed by replating the cells in various combinations 24 h post-transfection (n = 3). Scalebar = 10 μm; average +/−SEM is shown; ***p < 0.001; **p < 0.01; *p < 0.05.

As we intended to use various Tau constructs, we first verified their expression and localization in cells. HEK293T cells were transiently transfected with non-tagged Tau and GLuc-tagged forms of Tau and TauE14. TauE14 is a pseudohyperphosphorylated mutant carrying 14 phosphomimetic (serine/threonine to glutamate) mutations^22^, which mimic hyperphosphorylation, a known driver of Tau misfolding and aggregation in AD and other tauopathies. Western blot analysis showed that these constructs are expressed at comparable levels in HEK293T cells (Fig. 1A). When transiently transfected, wild-type Tau constructs did not promote SG formation and associate with SGs, as shown by co-immunostaining with Tau-5 and TIA-1 antibodies (Fig. 1B). Cells transfected with TauE14 showed a few puncta that co-stained with Tau and TIA-1, while cells expressing Tau-GLuc treated with arsenite, a classical inducer of SGs, showed a prominent stress granule response and also some recruitment of wildtype Tau to SGs (Fig. 1B,C).

Although arsenite induces a robust SG response – all cells resulted positive for TIA-1-positive SGs – it is toxic to cells via multiple mechanisms. In further experiments, we used salubrinal, an inhibitor of eIF2α dephosphorylation^23^, to promote SG formation with less toxicity^24^ (Fig. 1D). Assessment of cell viability with a resazurin reduction assay that monitors cellular metabolism showed that a 4-hour treatment with 100 μM salubrinal did not significantly decrease viability compared to the untreated control cells. On the other hand, Tau transfection itself resulted in almost 50% decreased viability with both the wildtype and the TauE14 forms, while further salubrinal treatment did not further exacerbate Tau toxicity, suggesting that SG formation does not affect viability in Tau-transfected cells (Fig. 1D). When cell viability was assessed by LDH release measurement, neither salubrinal or arsenite treatments increased LDH release, suggesting that the cell membrane remained intact in the experimental conditions used in this study (Fig. 1E). However, expression of Tau or TauE14 increased LDH release. Thus, both viability assays used, resazurin reduction and LDH release assays, showed similar effects on cell viability.

As a previous study suggested that TIA-1 directly interacts with Tau^17^, we next used a reversible protein-fragment complementation assay (PCA) to study the interaction of TIA-1 with Tau and TauE14 in live cells. Complementary fragments of Gaussia luciferase (GLuc) were tagged to TIA-1 and Tau, and the TIA-1-GLuc1, Tau-GLuc2 and TauE14-GLuc2 constructs were coexpressed in HEK293T cells. Generation of luciferase is only possible if the proteins come to close proximity (<10 Å) with each other allowing folding the GLuc fragments into active luciferase. As shown in Fig. 1F, when coexpressed wildtype Tau shows only weak interaction with TIA-1 while TauE14 shows more than 6-fold higher interaction signal. In agreement with the previous study^17^, the increased PCA signal level for TauE14/TIA-1 suggests enhanced interaction for the (pseudo)hyperphosphorylated Tau with TIA-1 compared to wildtype Tau.

In the light of the emerging literature of cell-to-cell propagation of Tau, we next asked if internalized extracellular Tau could associate with SGs. For this purpose, we generated separate batches of cells expressing single reporter fragments: TIA-1-GLuc1, Tau-GLuc2, TauE14-GLuc2 or the control plasmid GSK3β-GLuc2. Cells expressing TIA-1-GLuc1 were then coplated together with an equal number of separately transfected cells expressing Tau-GLuc2, TauE14-GLuc2 or GSK3β-GLuc2, and bioluminescence was measured after 24 hours. Generation of luminescence signal would thus be only possible if one of the expressed reporter proteins was capable of transferring to neighboring cells. While in wells containing cells expressing TIA-1-GLuc1 and GSK3β-GLuc2 the signal was nearly undetectable, there was clear luminescence signal generated in wells containing cells expressing TIA-1-GLuc1/Tau-GLuc2 and TIA-1-GLuc1/TauE14-GLuc2 combinations (Fig. 1G). This suggests that one of the reporter proteins had been secreted and internalized by the cells expressing the complementary reporter protein fragment. Interestingly, in the coplating assay wildtype Tau generated comparable signal to TauE14 suggesting that it either had better ability for cell-to-cell transfer or it had acquired a property that enhances its interaction with TIA-1.

**Figure 2:**
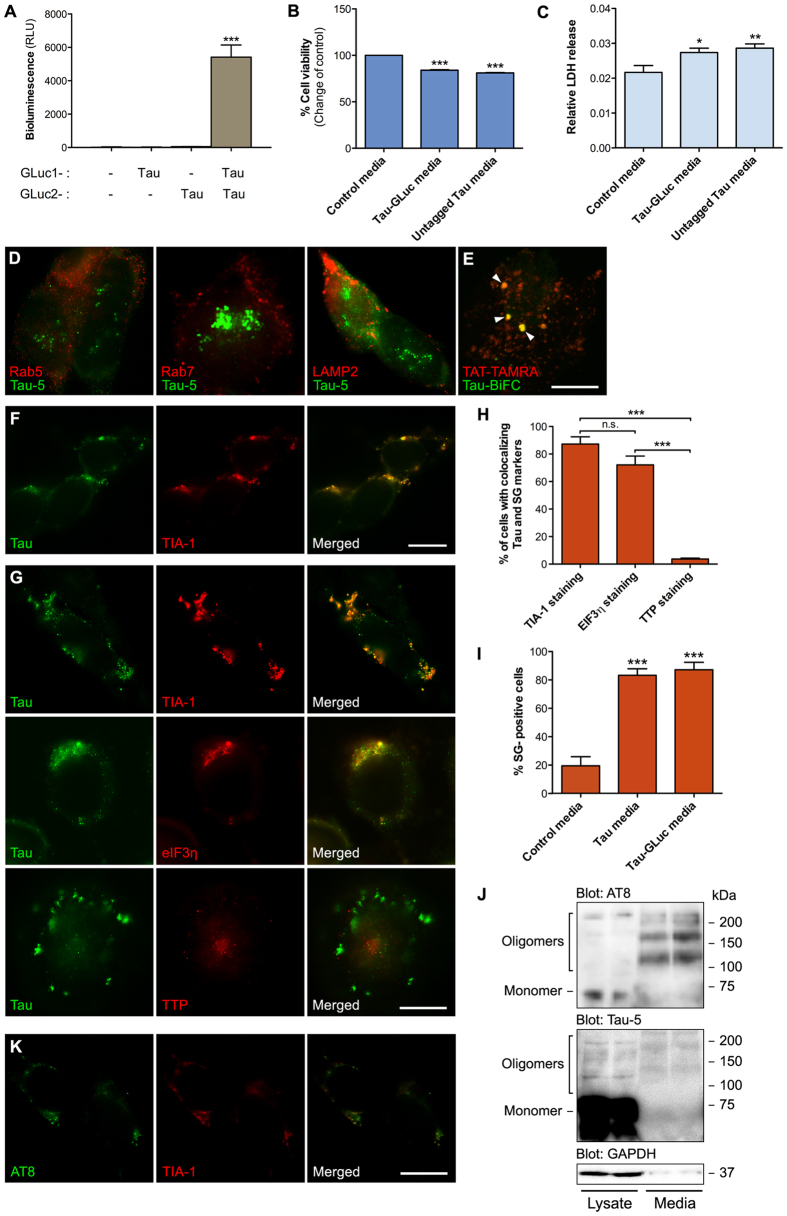
Internalized extracellular Tau localizes to stress granules. (**A**) PCA from conditioned media collected from HEK293T transiently transfected with either mock plasmid, Tau-GLuc1, Tau-GLuc2 or Tau-GLuc1/Tau-GLuc2. Secreted Tau dimers accumulate in fresh serum-free media (n = 3). Media was conditioned for 24 h with HEK293T cells expressing different forms of Tau for the subsequent experiments. (**B**) Resazurin-based cell viability assay showing decreased viability of cells exposed to Tau-GLuc1/2-containing media compared to the control-media exposed cells (n = 6). (**C**) Relative LDH release for cells exposed to Tau-GLuc1/2-containing media show slightly decreased viability compared to the control cells (n = 4). (**D**) Naïve HEK293T cells were exposed to Tau-GLuc conditioned media for 6 h and stained with Tau-5 and (from left to right) Rab5, Rab7 and Lamp2 antibodies to mark early endosomes, late endosomes and lysosomes, respectively. (**E**) Fluorescent TAT-TAMRA peptide was used as a marker for macropinosomes and visualized in live-cell imaging together with Tau-BiFC containing media, showing some colocalization. Tau-BiFC media was produced collecting conditioned media from HEK293T cells transfected with Tau-GFP10C and Tau-GFP11C, two Tau constructs carrying complementary fragments of GFP. Similar to GLuc-based PCA, generation of GFP signal requires Tau dimerization. (**F**) Naïve HEK293T cells were exposed to untagged Tau conditioned media for 6 h and stained with Tau-5 and SG marker TIA-1. (**G**) Naïve HEK293T cells exposed to Tau-GLuc1/2 containing media for 6 h and costained with Tau-5 and SG markers TIA-1, eIF3η and TTP. (**H**) Quantitative analysis of SGs using different markers indicates that TIA-1 and eIF3η staining frequently localize with Tau while TTP colocalization with Tau is found in very few cells (n = 4). (**I**) Quantitative analysis of TIA-1-positive SG formation in cells conditioned with Tau-GLuc and Tau media compared to control media (n = 4). (**J**) Western blot of Tau-GLuc-transfected cell lysate and Tau-GLuc conditioned media, stained with AT8, Tau-5 and GAPDH antibodies. Media was concentrated by 250x using filter centrifugation before loading on gel. The blot picture was cropped from a larger image, maintaining all the stained bands. (**K**) Naïve HEK293T cells were exposed to Tau-GLuc conditioned media for 6 h and stained with AT8 and TIA-1 antibodies, showing colocalization of Ser199/Ser202/Thr205-phosphorylated Tau and TIA-1 in the recipient cells. Scalebar = 10 μm; average +/−SEM is shown; ***p < 0.001; **p < 0.005; *p < 0.05.

We recently developed a PCA-based method for monitoring cell-to-cell transfer of Tau dimers in live cells^25^. As shown in Fig. 2A, luminescence-emitting Tau dimers are effectively secreted by HEK293T cells when Tau-GLuc1 and Tau-GLuc2 reporters are coexpressed. When naïve cells were exposed to either mock-transfected, Tau-GLuc1/2 or untagged Tau conditioned media, there was a mild but significant decrease (−20%) in cell viability (Fig. 2B,C), suggesting that the secreted Tau species (both GLuc-tagged and untagged native Tau) are similarly toxic to the cells.

Since the extracellular Tau was internalized and localized to punctate structures in the recipient cells (Fig. 2D–G), we used a panel of markers of cytosolic vesicles to identify the structures where internalized Tau localizes. Six-hour exposure of naïve recipient cells to Tau-GLuc1/2 or untagged Tau conditioned media resulted in similar punctate Tau-5 staining inside the cells (Fig. 2D–G). Interestingly, there was no colocalization of Tau-5 with Rab5 (marker for early endosomes), Rab7 (marker for late endosomes) or Lamp2 (marker for lysosomes) (Fig. 2D). As macropinosomes were recently proposed as a major route of cellular entry for Tau^20^, we used a fluorescently labeled TAT-TAMRA peptide as a marker of macropinosomes. Since TAT-TAMRA works only in live-cell imaging, we used Tau-BiFC dimer-conditioned media instead of Tau-GLuc1/2. The split GFP system works similarly to GLuc-based PCA but the refolded GFP reporter may be less reversible^25^,^26^. For a qualitative localization study, the combination of Tau-BiFC with TAT-TAMRA was thus justified. As shown in Fig. 2E, cells exposed to Tau-BiFC-conditioned media show colocalization of GFP signal with TAT-TAMRA suggesting that Tau enters HEK293T cells via macropinosomes. However, it should be noted that this does not exclude the possibility that other routes of entry may contribute to internalization of Tau.

As only minority of Tau-positive structures in recipient cells showed colocalization with vesicular structures, we reasoned that the rest of the cytosolic punctate staining might reflect the localization of internalized Tau with non-vesicular cellular bodies, particularly stress granules. Immunofluorescence staining revealed a significant colocalization of untagged Tau (Fig. 2F) and Tau-GLuc1/2 (Fig. 2G) with the SG marker TIA-1. Similar colocalization was also found with another SG marker eIF3η but not with TTP (Fig. 2G). While roughly 70–90% of cells exposed to Tau conditioned media showed SGs that costained for Tau and TIA-1 or eIF3η, only 4% of cells contained SGs positive for Tau and TTP (Fig. 2H). Tau internalization also clearly promoted stress granule formation in the recipient cells, as only 20% of HEK293T cells exposed to control media contained TIA-1-positive SGs while > 80% of cells exposed to Tau-GLuc1/2 or Tau-conditioned media contained SGs (Fig. 2I).

Because of the differences seen between wildtype Tau and TauE14 in the transfection and the coplating conditions (Fig. 1F–G), we hypothesized that wildtype Tau could undergo posttranslational modification (such as phosphorylation, as suggested by the phosphomimetic form of Tau) that would enhance its ability to interact with proteins involved in SG formation. To test this hypothesis, we concentrated Tau conditioned media and analyzed it by Western blotting together with the cell lysate. As expected based on previous results, the cell lysate contained the vast majority of total Tau protein (Tau-5 staining in Fig. 2J). However, there was a remarkable enrichment of Tau species phosphorylated at the AT8 epitope in the conditioned media as compared to the lysate (AT8 staining in Fig. 2J). Furthermore, the AT8 phosphorylated secreted Tau species were mostly SDS-stable oligomers, which were nearly absent from the cell lysate. Immunofluorescence staining of cells exposed to Tau conditioned media showed that internalized Tau associated with SGs was phosphorylated at the AT8 epitope (Fig. 2K).

**Figure 3:**
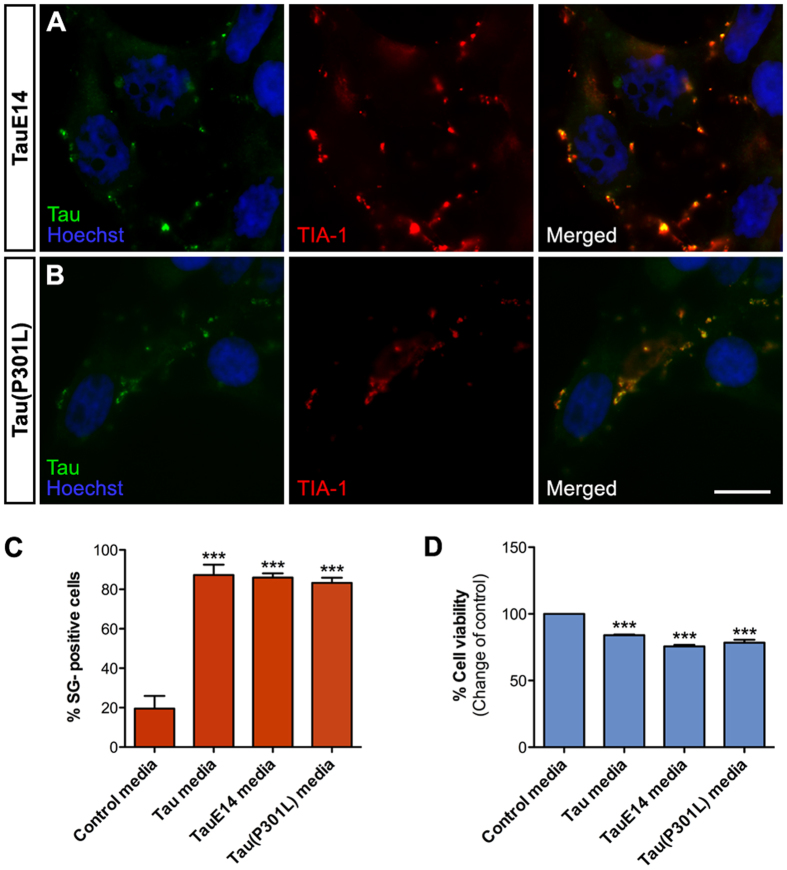
Internalized TauE14 and Tau(P301L) localize to stress granules. (**A**) Naïve HEK293T cells were exposed to TauE14-GLuc media for 6 h and stained with Tau-5 and TIA-1 antibodies. (**B**) Similarly to panel A, cells were exposed to Tau(P301L)-GLuc conditioned media and stained with Tau-5 and TIA-1 antibodies. (**C**) Quantitative analysis of TIA-1-positive SGs in cells conditioned with Tau-GLuc media, TauE14-GLuc and Tau(P301L)-GLuc conditioned media compared to control media (n = 4). (**D**) Resazurin-based cell viability assay for cells exposed to Tau-GLuc, TauE14-GLuc and Tau(P301L)-GLuc conditioned media (n = 4). Scalebar = 10 μm; average +/−SEM is shown; ***p < 0.001; **p < 0.01.

In the light of these findings, we also tested if other modifications in Tau affect its SG recruitment. As shown in Fig. 3A,C, internalized TauE14 was recruited to SGs and also reduced viability of recipient cells. P301L mutation, which causes familial frontotemporal dementia via increased Tau aggregation^27^,^28^, also localized to SGs after internalization from the conditioned media (Fig. 3B,C). In HEK293T cells, we did not observe significant quantitative or morphological differences between the SG association of wildtype Tau, TauE14 or Tau(P301L).

**Figure 4:**
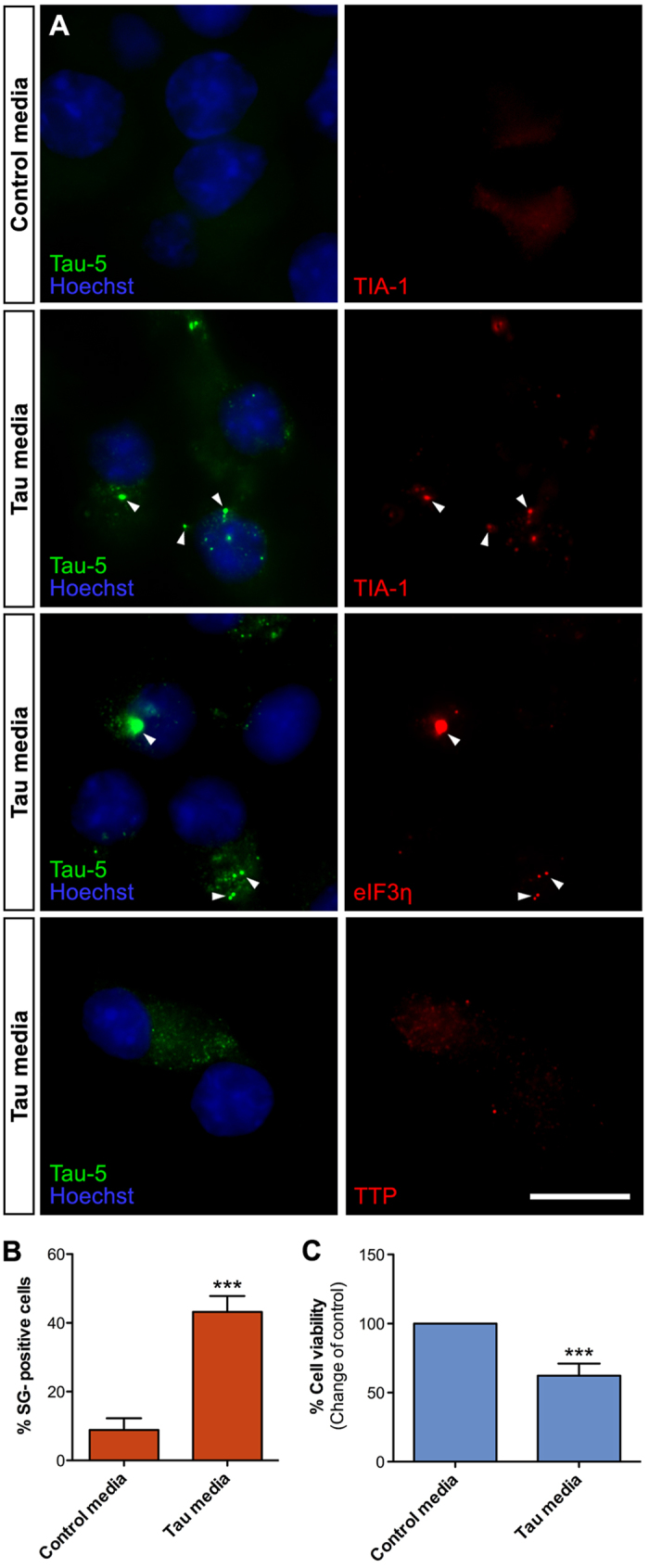
Internalized Tau is recruited to stress granules in N2A cells. (**A**) Naïve N2A cells exposed to Tau-GLuc conditioned media for 6 h and stained with Tau-5 and stress granule markers TIA-1, eIF3η and TTP. TIA-1 and eIF3η show clear colocalization with internalized Tau, while TTP does not show any. (**B**) Quantitative analysis of SG formation in cells exposed to Tau conditioned media compared to control media indicates that about 40% of N2A cells contained TIA-1-positive SGs following Tau uptake (n = 4). (**C**) Resazurin-based cell viability assay indicates that viability of N2A cells exposed to Tau-GLuc media was significantly decreased compared to cells exposed to control media (n = 8). Scalebar = 10 μm; average +/−SEM is shown; ***p < 0.001.

Next, we examined if Tau uptake and recruitment to SGs occur also in N2A neuroblastoma cells. Figure 4A shows that internalized Tau is recruited to SGs positive for markers TIA-1 and EIF3η, but not with TTP, in N2A cells. Interestingly, while in HEK293T cells, nearly 90% of the cells were positive for TIA-1/Tau staining, only 40% of N2A cells displayed Tau-positive SGs (Fig. 4B). Also, we observed fewer but occasionally larger SGs per cell in N2A cultures as compared to HEK293T cells. Despite these differences, the viability of N2A cells was significantly decreased (−38%) following exposure to Tau conditioned media (Fig. 4C). These data suggest that, despite minor differences uptake efficiency and SG morphology, different cell types uptake extracellular Tau resulting in its recruitment to SGs and that this is associated with reduced cell viability. Moreover, hyperphosphorylation-induced oligomerization may be an important factor promoting the SG recruitment of internalized Tau species.

### *TIA-1* is required for stress granule recruitment of internalized Tau

**Figure 5:**
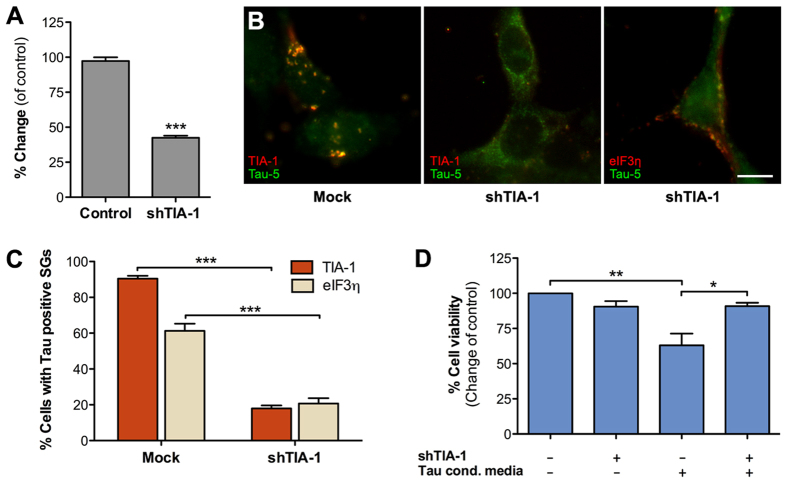
Tau localization to stress granules requires TIA-1. (**A**) TIA-1 shRNA knockdown efficiency determined by qPCR following shRNA plasmid transfection of HEK293T cells. Levels of TIA-1 mRNA were normalized to GAPDH mRNA levels (n = 2). (**B**) TIA-1 was transiently knocked down in HEK293T cells (middle and right images) that were then exposed to Tau-GLuc-conditioned media and stained with Tau-5 and stress granule marker antibodies. The middle image shows typical cells with mostly cytosolic, non-punctate staining of Tau. The image on the right shows an example of a cell with SGs costaining with Tau and eIF3η. (**C**) Quantitative analysis of Tau-positive stress granule formation. SGs were present in the majority of cells exposed to Tau media when TIA-1 is normally expressed but were significantly decreased when TIA-1 was knocked down. Similar results were obtained with both TIA-1 and eIF3η staining (n = 3). (**D**) Resazurin-based cell viability assay showed that TIA-1 knockdown had no effect on cell viability *per se* but was able to improve viability in cells exposed to Tau-GLuc-conditioned media (n = 3). Scalebar = 10 μm; average +/− SEM is shown; ***p < 0.001; **p < 0.01; *p < 0.05.

Next, we further investigated the role of TIA-1 in the SG recruitment and the associated toxicity of Tau. First, we used RNAi to silence TIA-1 expression in recipient cells. Knockdown efficiency of TIA-1 shRNA plasmids (shTIA-1), determined using qPCR was about 50% compared to the mock-transfected cells expressing endogenous levels of TIA-1 (Fig. 5A). Recipient cells transfected with shTIA-1 and exposed to Tau-GLuc conditioned media showed a remarkable reduction in TIA-1-positive SGs and recruitment of Tau reporters to the SGs (Fig. 5B,C). When TIA-1 was only partially expressed, the there were significantly less cells containing Tau-positive SGs compared to control-transfected cells exposed to Tau conditioned media (Fig. 5C). Notably, Tau-5 staining indicated that Tau was internalized from the media but its distribution appeared to be less punctate and more evenly spread throughout the cytoplasm of the shTIA-1 cells. Immunostaining of shTIA-1-transfected cells with eIF3η showed that SG-like structures were still forming in the cells independently of the presence of TIA-1, and some cells contained Tau-positive SGs that colocalized with eIF3η (Fig. 5B, right image). However, the number of SGs that costained for Tau/TIA-1 and Tau/eIF3η was reduced by 80% and 66%, respectively, compared to the mock-transfected cells (Fig. 5C). Importantly, partial absence of TIA-1 also protected recipient cells from Tau toxicity as shown by improved viability after exposure to Tau-GLuc conditioned media (Fig. 5D), in line with a recently published study^29^. Altogether these data suggest that TIA-1 plays a major role in recruitment of internalized Tau to SGs and also modulates the toxicity of propagating Tau species.

### Internalized Tau alters stress granule dynamics and sensitizes cells to stress

**Figure 6:**
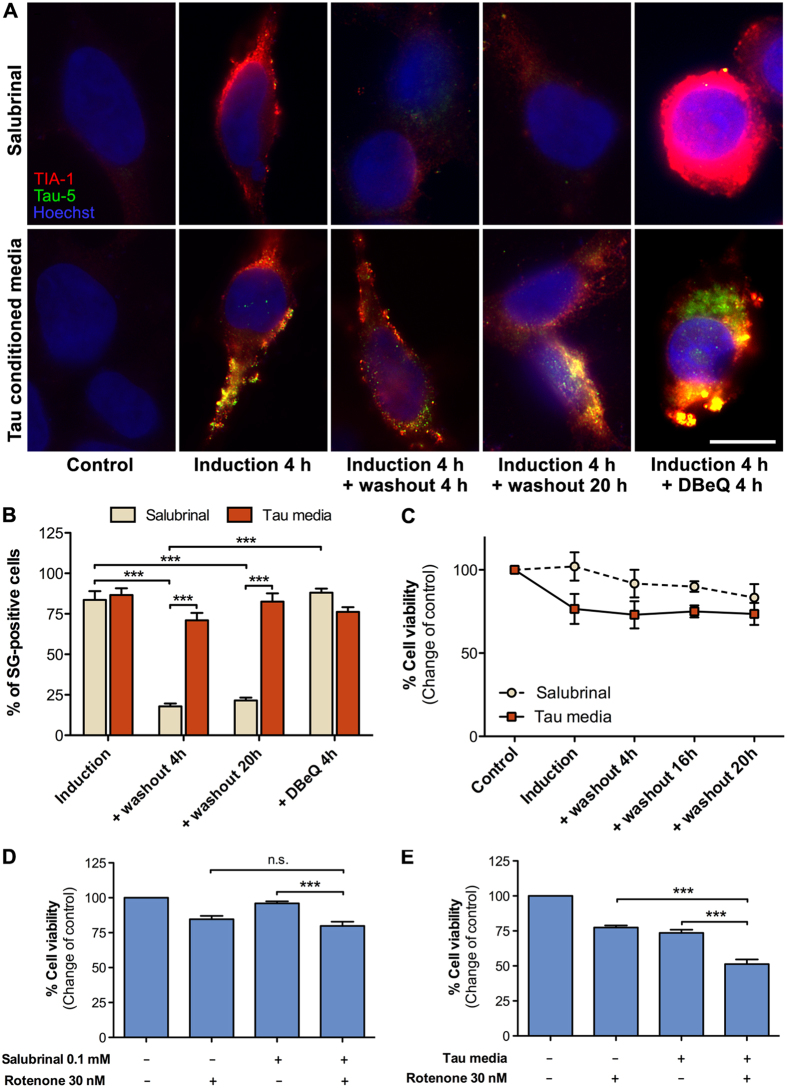
Internalized Tau alters stress granule dynamics and sensitizes cells to stress. (**A**) Tau-GLuc-conditioned media or 0.1 mM Salubrinal was applied to HEK293T cells for 4 h, followed by a washout with normal media. Cells were fixed at 4 and 20 h after the washout and stained with TIA-1 and Tau-5 antibodies. DBeQ (10 μM) was used as a positive control to inhibit stress granule disassembly after the treatments. (**B**) Quantitation of immunofluorescence images: stress granule-positive cells over total cells were counted for each condition (n = 3). (**C**) Cell viability assay for the washout experiment (n = 4). (**D,E**) Cell viability assay for cells treated either with salubrinal (**D**) or with Tau-conditioned media (**E**) followed by 4 hours of recovery in fresh media and further treatement with 30 nM Rotenone for 6 h. In the salubrinal control cells Rotenone showed minimal toxicity while in cells exposed to Tau-GLuc-conditioned media Rotenone significantly decreased cell viability (n = 4). Scalebar = 10 μm; average +/− SEM is shown; ***p < 0.001.

As abnormal SG function and dynamics have been implicated to contribute to pathogenesis of neurodegenerative diseases, we investigated the role of internalized Tau in SG clearance and dynamics. SGs normally resolve within a few hours from the disappearance of the stressful stimulus that promoted their formation^12^. When induction of SGs with 4 h treatment with salubrinal was followed by a 4-hour washout period, the SGs had nearly completely disappeared from the cytoplasm (Fig. 6A). However, in the presence of DBeQ, an inhibitor of VCP-mediated SG clearance^15^, the SGs were not effectively resolved. When the recipient cells were exposed to Tau-GLuc conditioned media for 4 hours, followed by 4 hours washout period with fresh, non-Tau-containing media, the SGs were still present. Even after 20 h washout, there was a significant amount of SGs positive for both Tau and TIA-1 present in the cells (Fig. 6A,B). Addition of DBeQ further accentuated the SG phenotype resulting in very large Tau/TIA-1 positive clusters.

Surprisingly, when viability of Tau-exposed and washed cells was measured, significant increase in cell death was not observed, even after the 20 h washout period (Fig. 6C). We hypothesized that the while the prolonged presence of SGs may not directly impair cell viability, the cells may be more sensitive to secondary stress. Cells that had been first pretreated with Tau-GLuc conditioned media for 4 hours, followed by a 4-hour recovery period and then incubated with subtoxic concentration (30 nM) of rotenone^30^, a mitochondrial complex 1 inhibitor, showed a significant decrease in cell viability (Fig. 6E). Notably, similar effect was not observed in salubrinal-pretreated cells (Fig. 6D). The reduction in cell survival of Tau-exposed cells was stronger compared to non-Tau-pretreated cells receiving rotenone or to cells treated with Tau-conditioned media only. Altogether this implies that Tau recruitment to SGs strongly affects their normal clearance dynamics and that while prolonged presence of SGs in cells might not directly affect viability in short-term, it might impair the ability of cells to cope with subsequent stressful events.

## Discussion

Many previous studies have established the ability of misfolded, pathological Tau forms to spread in a time-dependent manner to neuroanatomically connected unaffected brain areas^31^,^32^,^33^. Tau transmission has been reported to occur transsynaptically from one neuron to another^34^ and be facilitated by microglial packing of Tau into exosomes^35^. Very little is currently known about the cellular mechanisms that are involved in cell-to-cell transfer of Tau or the events and functional consequences triggered by its internalization. Existence of Tau strains with different cell-to-cell propagation properties has been recently reported^36^,^37^. The variety arising from Tau splicing and the numerous posttranslational modification sites suggest that different Tau strains may have different properties and could propagate differently through different non-exclusive mechanisms.

A previous study showed that SG proteins TIA-1 and TTP bind to phospho-Tau and localize to neurofibrillary tangles in the brains of late stage AD patients and animal models of FTDP-17^17^. It is currently not clear whether TIA-1 and TTP interact with Tau under normal physiological conditions or are these interactions limited to pathological conditions. The experimental conditions used in our current study, based on Tau overexpression and resulting in secretion of hyperphosphorylated, oligomeric Tau, are more likely to mimic a pathological setting than normal physiological conditions. Thus, our *in vitro* results shed more light on how the pathological Tau species affect the viability of cells that internalize Tau, and provide further evidence for the role SGs in propagation of pathology in tauopathies. While this work was under review, a separate study was published showing that TIA-1 and Tau act synergistically to modulate degeneration of neurons, and that TIA-1 knockdown or knockout inhibits Tau misfolding and toxicity^29^.

Internalized Tau, unlike overexpressed cytosolic Tau, promotes SG formation despite the much higher abundance of Tau in the cytosol. Our data indicates that modifications in Tau composition or structure have occurred during the secretion-uptake process, and that only the internalized Tau species (or some of them) are able to effectively interact with TIA-1 and promote formation of SGs. The role of TIA-1 seems critical as even a partial knockdown strongly reduced Tau recruitment to SGs, without affecting internalization of Tau. Interestingly, the ability of wildtype Tau to interact with TIA-1 was significantly enhanced after internalization as compared to cytosolic, transfected Tau, if considering the relative abundance of the two proteins in the two conditions. Instead, TauE14, carrying 14 Ser/Thr-to-glutamate mutations mimicking hyperphosphorylation of Tau, displayed increased affinity towards TIA-1 inside the cells, further supporting the idea that phosphorylation regulates the interaction of TIA-1 and Tau. The interaction between TIA-1 and Tau seemed specific, as another classical SG marker, TTP, previously shown to interact with Tau *in vivo*^17^, did not colocalize with Tau-positive SGs in either HEK293T or N2A cells. It is possible that, among the variety of proteins that constitute differentially composed SGs, TTP is not primarily recruited to Tau-induced SGs. Moreover, there may be cell-type differences in the sets of SG proteins involved or that recruitment of TTP to neurofibrillary tangles is part of a different cellular process that was not replicated in our *in vitro* system.

Interestingly, wildtype Tau, the pseudohyperphosphorylated form TauE14 and the disease-linked mutant Tau(P301L) appear to behave in a similar way when internalized from the media, suggesting that either the differences between these three forms of Tau are not relevant for the uptake process and recruitment to SGs, or that during secretion wildtype Tau acquires novel features that promote its localization to SGs. This last hypothesis is supported by the result that secreted wildtype Tau was heavily phosphorylated at the AT8 epitope and considerably more oligomeric compared to intracellular Tau. Although there may be other posttranslational Tau modifications involved, the evidence presented here suggests that hyperphosphorylation may be associated with the recruitment of wildtype Tau to SGs following internalization. Interestingly, it appears that either Tau hyperphosphorylation is coupled to the secretion process or that these cells preferentially direct those Tau species that are heavily phosphorylated for secretion.

In our current study, internalized Tau colocalized with the macropinosome marker TAT-TAMRA, similarly to a previous report^20^, but did not colocalize with markers of early and late endosomes or lysosomes. Macropinosomes have recently been implicated in neurodegenerative diseases as they allow the intake of large structures such as prion-like protein aggregates (reviewed in^38^). It is likely that in our system macropinosomes represent the internalization mechanism of only a portion of the total Tau entering the cell. Currently, the mechanism of how Tau escapes from the macropinosomes to the cytosol, a prerequisite for interaction with SG proteins, remains unknown. Interestingly, Tau oligomers seem to be highly surface active and may be able to disrupt the vesicle membrane, which could explain their escape from vesicles to the cytosol^21^,^39^.

One current hypothesis for the role of SGs in neurodegenerative diseases considers them as a pathological seeding point, where misfolded proteins find other low complexity proteins with aggregating domains, forming a nucleating core for further aggregation^40^,^41^. Several recent reports have provided further support for this hypothesis by showing that RBPs FUS and hnRNPAs undergo phase transition between a liquid phase, in which the proteins organize themselves into droplets, and a solid phase, which promotes aggregation and fibril assembly^42^,^43^,^44^. Interestingly, low complexity sequences, a common feature of both Tau and TIA-1, have a flexible secondary structure, but in presence of a ligand the structure becomes more rigid, possibly promoting fibrillization^45^. These nuclei that would act as a pathological seed would not behave as physiological SGs, therefore they would fail to be degraded and resolved, prolonging their presence in the cytoplasm. In this study, we report that following removal of the Tau-containing media and allowing the cells to recover from stress, persistent Tau-containing SGs were still detected in cells for at least up to 20 hours (longer timepoints not tested), suggesting that Tau-induced SGs and salubrinal-induced SGs are functionally and possibly structurally different, and undergo different clearance kinetics. Such abnormally long-lasting SGs were associated with sensitization of cells to secondary stress. This could loosely mimic an AD or any other neurodegenerative disease scenario, where the proteinopathy and progressive loss of neurons develops slowly over time^46^, as do the clinical symptoms.

Our current data suggests that SGs may play a central role in cell-to-cell propagation of Tau species, possibly serving as a pathological seeding point. While this study describes a new cellular mechanism for the toxicity and propagation of internalized Tau through alteration of SG dynamics, future studies are required to elucidate the exact mechanisms underlying Tau recruitment to SGs from macropinosomes and whether there are specific Tau species with a particular molecular signature that preferentially promote SG formation.

## Materials and Methods

### Constructs

The split *Gaussia princeps* luciferase (GLuc) system used in this study has been previously described^47^. The original humanized GLuc plasmids were a gift from Prof. Stephen Michnick (Université de Montréal, Montreal, Canada). The cDNAs for Tau (human isoform 0N4R; GeneBank accession number BC114948) and TIA-1 cDNA (BC046812.1) were purchased from Thermo Scientific. TauE14 (the pseudohyperphosphorylated form of Tau) plasmid^22^ was a kind gift from prof. Mel B. Feany (Harvard Medical School, Boston, MA, USA). Tau(P301L) mutant was a gift from Dr. Karen Ashe (University of Minnesota, Minneapolis, MN, USA) (Addgene plasmid #46908). The hGLuc fragments were cloned to C-termini of Tau, TauE14, Tau(P301L) and TIA-1, separated by a (GGGGS)_2_SG linker in a pcDNA3.1/zeo plasmid backbone.

Pair of commercial plasmids containing the parts of the split GFP was used as the basis for cloning the bimolecular fluorescence complementation (BiFC) assay plasmids (Sandia Biotech Inc., USA)^48^. The BiFC assay is based on splitting superfolder GFP into two complementary fragments encoding β-strands 1–10 (1–214 aa) and the 11^th^ β-strand (215–230 aa). The GFP fragments were cloned to the Tau C-terminus.

The TIA-1 shRNA clones (TRCN0000074463, TRCN0000074464, TRCN0000074465, TRCN0000074466, TRCN0000074467; in pLKO.1 plasmid) were acquired from the TRC1.0 library via the Functional Genomics Unit Biomedicum Helsinki. All plasmids were sequenced to confirm their identity. The clone TRCN0000074465 was used in the knockdown experiments.

### Chemicals

Classic stress granule inducer (sodium arsenite), EIF2α inhibitor (salubrinal) and p97/valosin-containing protein (VCP) inhibitor (DBeQ) were purchased from Sigma-Aldrich and used at concentration of 500 μM, 100 μM and 10 μM, respectively. Rotenone was a kind gift from Dr. Timo Myöhänen (Faculty of Pharmacy, University of Helsinki) and used at concentration of 30 nM.

### Cell culture and media production

Human embryonic kidney (HEK) 293 T cells were cultured in DMEM supplemented with 1% (v/v) penicillin, streptomycin and L-glutamine (Lonza) and 10% (v/v) FBS (Invitrogen) at 37 °C and 5% CO_2_ atmosphere. For Tau conditioned media production, cells were plated at a density of 9 × 10^6^ cells in 10 cm plates. Transfection was performed using JetPei (Polyplus) according to manufacturer’s instructions. The culture medium was replaced 24 hours post-transfection with phenol red-free DMEM (Invitrogen) supplemented with 1% (v/v) penicillin and streptomycin. The medium was collected 48 hours post-transfection. Detached cells and cell debris was removed by centrifugation for 10 minutes at 4000 × g and medium was stored at −20 °C. Before loading on SDS-PAGE gels, the media samples were concentrated using Amicon filters with 30 kDa cut-off (Millipore) according to manufacturer’s instructions.

### Protein-fragment complementation assay (PCA)

HEK293T cells were plated at a density of 10 000/well on 96-well plates with white walls (Perkin Elmer) and transiently transfected with 100 ng DNA per well of phGLuc(1 C) and phGLuc(2 C) expression plasmids or mock. The experiments were performed 48 h post-transfection: cells were washed with warm PBS and the media was replaced with Tau-GLuc conditioned or mock-conditioned media. Salubrinal was added to the medium where indicated. PCA luminescence signal detection was performed with Victor^35^ (Perkin Elmer) or Varioskan (Thermo Scientific) plate reader following well-by-well injection of the hGLuc substrate native coelenterazine (NanoLight Technology, USA). Eight replicate wells were analyzed per condition and at least three independent replicate experiments were performed.

### Live-cell imaging

HEK293T cells were incubated for 4 hours with Tau-BiFC containing media, and for the last hour 10 μM of TAT-TAMRA peptide (Anaspec) was added to the media where indicated. Just before imaging, cells were washed and media was replaced by phenol red-free DMEM supplemented with 15 mM Hepes buffer (Life Technologies). Imaging was performed with a Zeiss LSM 710 upright confocal and 63x water immersion objective at stable 37 °C temperature. Images were analyzed with ImageJ software.

### Immunofluorescence imaging

Immunofluorescence imaging was performed as previously described^30^. Briefly, cells were grown on poly-L-lysine (Sigma) coated coverslips and fixed with 4% PFA in PBS for 20 minutes. Coverslips were washed with PBS and incubated for 1 hour with blocking buffer (1% BSA, 0.1% gelatin, 5% goat serum, 0.1% Triton X-100 and 0.05% Tween-20 in PBS; all from Sigma). Coverslips were then incubated with primary antibodies diluted 1:500 in 1% BSA and 0.1% gelatin in PBS overnight at +4 °C. The primary antibodies used were: Tau-5 (Invitrogen, mouse, 1:600), TIA1 (Santa Cruz, goat, 1:500), eIF3η (Santa Cruz, goat, 1:500), TTP (Santa Cruz, rabbit, 1:300), Rab7 (Cell Signaling, rabbit, 1:400), Rab5 (Novus Biological, rabbit, 1:500), Lamp2 (Sigma, rabbit, 1:500) and AT8 (Thermo Scientific, mouse, 1:500). The Alexa-Fluor conjugated secondary antibodies (Invitrogen) were incubated for 1 hour at room temperature in dilution 1:2000. The secondary antibodies used were 488-goat-anti-mouse, 568-donkey-anti-goat and 568-donkey-anti-rabbit. Nuclei were stained with Hoechst 33342 (Invitrogen) and coverslips mounted on microscope glasses with ProLong Gold anti-fade reagent (Invitrogen). Pictures were taken with a Zeiss AxioImager M1 epifluorescence microscope. Raw images were compiled using Adobe Photoshop. Quantifications from immunofluorescence images were done by counting stress granule-positive cells per total number of cells in randomly selected fields. Each field contained at least 50 cells and at least three images per condition were analyzed.

### Cell viability assays

Resazurin sodium salt (Sigma) was diluted in phenol red-free DMEM and added to the cells at the concentration of 100 μM. Cells were incubated in +37 °C with 5% CO_2_ for 2 hours. Fluorescence was measured with Victor^35^ plate reader for 0.1 second at 530 nm excitation and 590 nm emission filters. Cell viability was calculated as percentage in comparison to a vehicle treated control.

LDH release was measured with CitoTox 96 Non-Radio Cytotoxicity Assay (Promega) according to manufacture’s instructions. Absorbance was detected at 490 nm with Victor^35^ plate reader. Relative LDH release is a ratio of LDH in the media versus total LDH both in the cell and the media. Eight replicate wells analyzed per condition and at least three independent replicate experiments were performed.

*qPCR* – The gene silencing effect of the shTIA-1 plasmids was assessed by qPCR. Primers were designed with Geneious software and synthesized by Oligomer Oy (Helsinki, Finland). The primers used were: for TIA1 (forward) 5′-ACAGCAGAACAAAGGAACCC-3′, (reversed) 5′-TGTCTGTTTCCTTGCTGGTT-3′; for GAPDH, (forward) 5′-ACCCCTTCATTGACCTCAACTACATGG-3′, (reversed) 5′-ATCCACAGTCTTCTGGGTGGCA-3′. RNeasy kit (Qiagen) was used to extract RNA from cell lysates, while RevertAid First Strand cDNA synthesis kit (Thermo Scientific) was used to translate RNA samples into cDNA, both according to manufacturer’s instructions. SYBR Green Maxima kit (Thermo Scientific) and RT-PCR cycler (Bio-Rad CFX95) were used to detect the relative mRNA amounts. Relative mRNA levels were calculated using the 2^ΔΔCT^ method in Microsoft Excel.

### Western blot

Western blotting was done as previously described^49^. Cells grown on poly-L-lysine-coated 6-well plates and transfected with 3 μg of total DNA per well were washed twice with ice-cold PBS 48 hours post-transfection, and extracted with extraction buffer [10 mM Tris-HCl, 1 mM EDTA, pH 6.8, 150 mM NaCl, 1% Triton X-100, 0.25% Nonidet P-40, Protease and Phosphatase Inhibitor cocktail tablets (Roche Molecular Biochemicals), 1 μM NaF]. Protein concentration of extracts was determined by BCA Protein Assay kit (Thermo Scientific) and equal amounts of cell lysates were resolved on 4–12% gradient Bis-Tris gels (Novex, Invitrogen) under reducing conditions. Proteins were transferred to PVDF membranes (GE Healthcare) by using semidry blotting (Bio-Rad). Tau-5 antibody (Invitrogen), AT8 antibody (Thermo Scientific), GAPDH antibody (Millipore), horseradish peroxidase-conjugated secondary antibodies and ECL Western blotting detection reagent (Thermo Scientific) were used to detect chemiluminescence signal.

### Statistical analyses

Statistical analyses were performed in GraphPad Prism software using one-way ANOVA followed by Bonferroni post-tests. Error bars shown represent standard error of mean (SEM).

## Additional Information

**How to cite this article**: Brunello, C. A. *et al*. Internalized Tau sensitizes cells to stress by promoting formation and stability of stress granules. *Sci. Rep.* **6**, 30498; doi: 10.1038/srep30498 (2016).
